# miR-184-5p inhibits cell proliferation, invasion and predicts prognosis of clear cell renal cell carcinoma by targeting NUS1 dehydrodolichyl diphosphate synthase subunit: Results from large-scale comprehensive identification and validation

**DOI:** 10.7150/jca.58053

**Published:** 2022-02-21

**Authors:** Wangrui Liu, Chunguang Ma, Hong Xu, Lijun Wang, Wenhao Xu, Hailiang Zhang, Zhisu Wang, Jun Li, Ji Zhang, Xigao Liu, Shuai Zhao, Tao Wang

**Affiliations:** 1 Department of Interventional Oncology, Renji Hospital, Shanghai Jiao Tong University School of Medicine, Shanghai, China.; 2 Affiliated Hospital of Youjiang Medical University for Nationalities, Baise, 533000, China.; 3 Department of Urology, Fudan University Shanghai Cancer Center, Shanghai, 200032, China.; 4 Department of clinical medicine, Medical School of Nantong University.; 5 Department of Hepatobiliary Surgery, Tenth People's Hospital of Tongji University, Shanghai, China, 200072.; 6 Department of Hepatic Surgery, Eastern Hepatobiliary Surgery Hospital, Naval, Medical University, Shanghai, China, 200438.; 7 Department of Urology, Qilu Hospital of Shandong University, Jinan, Shandong, 250012, P.R. China.; 8 Department of Transplantation, Xinhua Hospital affiliated to Shanghai Jiao Tong University School of Medicine, Shanghai, China.

**Keywords:** Clear cell renal cell carcinoma, microRNA, NUS1, invasion, proliferation, biomarker

## Abstract

Clear cell renal cell carcinoma (ccRCC) has become a common malignant cancer with increasing incidence rate and high recurrence risk in genitourinary oncology around the world. Recently, miRNAs were identified to affect pathogenesis, development, molecular functions, and prognosis of ccRCC. In this study, microRNA-184-5p (miR-184-5p) was identified from three independent ccRCC cohorts and was determined as a significantly distinct prognostic biomarker. Relative miR-184-5p expression was found in A-498 and 786-O ccRCC cells compared with HK-2 cells. After ccRCC cells were transfected with miR-184-5p mimics or inhibitor, biological abilities of miR-184-5p in tumor cell proliferation, cycle, apoptosis and invasion were determined. Additionally, we confirmed the direct relationship between miR-184-5p and NUS1 dehydrodolichyl diphosphate synthase subunit (NUS1) by using the Luciferase reporter and rescue assays. These results indicated that the expression level of miR-184-5p in human ccRCC cells and tissues was reduced, and the up-regulation of miR-184-5p regulated A-498 and 786-O cell proliferation, invasion and apoptosis by directly targeting NUS1. These findings may provide new theoretical targets for treatment strategies and drug development of ccRCC.

## Introduction

Kidney cancer has become one of the most common urinary tumors in the world, with an estimated 73,820 new cases in the United States in 2019, of which 14,770 deaths 1. Previous studies have shown that an estimated 66,800 new cases and 23,400 deaths in China in 2015 2. Clear cell renal cell carcinoma (ccRCC) is the main and most common subtype of renal cell carcinoma (RCC) in adults. According to the World Health Organization, ccRCC is one of the tumors with the highest mortality of the urinary system, with approximately 90,000 mortality cases per year worldwide 3. Despite extensive researches into the mechanisms of carcinogenesis and aggressive progression, the etiology of ccRCC remains unclear. Until 2019, the development and progress of ccRT was reportedly linked to multiple factors, such as genetic aberrations, tumor micro environment, cellular and metabolic factors 4. Considering high morbidity and mortality of ccRCC, it is critical to reveal the potential treatment strategies as well as the underlying molecular mechanisms and to explore molecular biomarkers for early diagnosis, prevention and individual therapy.

MicroRNA (miRNA) is a type of non-coding RNA with a length of 18 to 22 nucleotides and it was firstly discovered in Caenorhabditis elegans in 1993^4^-^7^. As one of the most important non-coding RNAs, miRNAs are involved in different biological activities and target a wide range of genes. Among these biological processes in which miRNAs are involved, especially the carcinogenic process is the most important 8. Like other RNA molecules, transcription of these molecules is mediated by RNA polymerases (such as II and III) 9. The product of this enzyme is a larger primary transcript (pri-miRNA) with a cap at its 5' end and a poly A tail at its 3' end. A microprocessor complex contains two main components - the proteins DGCR8 and RNase Drosha as well as the cleaves pri-miRNA into a precursor miRNA (pre miRNA) with a stem-loop structure. Then, after the pri-miRNA is transported to the cytoplasm through the function of Ran/GTP/exportin 5 complex, it will be subjected to an enzymatic reaction catalyzed by the RNase III enzyme (Dicer) and generate unstable miRNA/miRNA duplexes. Select a substrate of this duplex as a mature miRNA molecule and direct it to the target messenger RNA through a protein complex called RISC. Then, miRNAs will interact with the 3′‐untranslated regions (3′‐UTR) of target mRNA and cause translational suppression or mRNA decomposition with regard to the grade of miRNA‐mRNA complementarity 10. Misregulation of miRNAs expression patterns has been reported to have considerable effects on cancer markers, such as inducing invasion and metastasis. The underlying mechanisms of this disorder include amplification or deletion of miRNA genes, imbalance of several important transcription factors (including p53 and c-Myc), epigenetic disorders such as insufficient methylation of the overall genomic DNA, and excessive tumor suppressor genes Methylation and impaired histone modification patterns, and defects in miRNA biogenesis 11. Some recent studies suggest that abnormal expression of miRNAs occurs in many types of cancer and may play a role in promoting or suppressing tumors 12, 13. However, direct assessment of microRNA-184 (miR-184) expression and its biological role are still rare in ccRCC.

With the development of biotechnology and computer technology, genome sequencing and bioinformatics have facilitated the investigation of the tumor transcriptome and proteome, which has advanced the current understanding of the underlying molecular mechanisms of ccRCC 14. In our study, we aimed to identify differential expressed hub miRNAs and hypothesized that the hub miRNA might play a vital role in tumorigenesis and dynamic process of tumor evolution. To test this hypothesis, we firstly identified the hub miRNAs using multiply datasets from public database and examined the expression levels of miR-184 in four renal cancer cell lines and a normal renal epithelial cell line. Subsequently, we attempted to explore the impact of miR-184 in the proliferation, migration and invasion of ccRCC cells. Overall, by using these methods, these results may provide new theoretical goals for treatment strategies for ccRCC.

## Materials and methods

### Screening for transcriptional microRNAs expression profiles

NCBI-GEO is regarded as a free public database of transcriptional expression profile and we obtained the differential expressed miRNAs (DEmiRNAs) profile from GSE12105, GSE55138 and GSE109368 in ccRCC and normal kidney tissues. The data of GSE12105 were obtained from the GPL6955 Agilent-016436 Human miRNA Microarray 1.0 (Feature Number version) and came from 12 kidney tumors and 12 normal kidney tissue samples. Similarly, the data of GSE55138 were based on the GPL14613 ([miRNA-2] Affymetrix Multispecies miRNA-2 Array). The gene microarray data were collected from 16 clear cell renal carcinoma and 9 adjacent non-tumor tissues. The GSE109368 data were obtained from the GPL18573 (Illumina NextSeq 500 (Homo sapiens)), including 12 primary ccRCC cancer tissues and 12 normal renal tissues.

### Standardization and elucidation of DEmiRNAs

DNA microarray analysis started with processing and standardization of raw biological data. This procedure eliminated noise from the biological data and guaranteed integrity. Then, background correction of probe data, standardization and summarization were performed by robust multiarray average analysis algorithm 15 in affy package of R software 15, 16.

The DEmiRNAs between normal kidney tissues and ccRCC samples were screened and identified across experimental conditions. Differential expressed miRNAs or genes were identified according to threshold of |LogFC|>1, P value<0.05. Delineating parameters such as adjusted P values (adj. P), Benjamini and Hochberg false discovery rate (FDR) and fold change were utilized to filter DEGs and to provide a balance between the discovery of statistically significant genes and limitations of false positives.

### Cell culture

The normal human proximal tubular epithelial cell line HK2 (ATCC, CRL-2190, RRID: CVCL_0302, Maryland, Rockefeller, USA) cells were maintained in DMEM/F12 medium (BasalMedia, #L310, Shanghai, China). The human ccRCC cell line A498, 786O, CAKI1, CAKI2 cells were obtained from National Collection of Authenticated Cell Cultures and maintained in RPMI 1640 (BasalMedia, #L210, Shanghai, China). All culture media were supplemented with 10% fetal bovine serum (FBS, Gibco, Shanghai, China), 2 mM L-glutamine, 100 U/mL penicillin and 89 mg/mL streptomycin (S2572, Selleck, China). Cells were cultured in a humidified incubator at 37 °C in a humidified atmosphere with 5% CO_2_.

### Total RNA extraction and quantitative real-time PCR analysis

For the detection of miRNA-184-5p expression, all miRNA extractions were performed using the miRNeasy Kit (Qiagen, Germany), resulting all in strict accordance with the manufacturer's instructions. miR-184-5p forward, 5′- GGTACCCAAGCAGATGGGATCCAAAGTTGGTG -3′ and reverse, 5′- CTCGAGGGCAAAGAAGTTTTGATGAAGCTTTG -3′. cDNA was synthesized using the miScript II RT Kit (Qiagen). The expression of miRNA was analyzed using the miScript SYBR Green PCR Kit (Qiagen) with RNU6 (miRNA) as an endogenous control. Total RNA from harvested cell lines was isolated by Trizol (Invitrogen, Carlsbad, CA), and qRT-PCR was performed using SYBR^®^ Premix Ex TaqTM (TaKaRa, Japan) according to manufacturer's protocol. The primers pairs were: NUS1 forward, 5′‐TGCCAGTTAGTAGCCCAGAAGCAA‐3′, NUS1 reverse, 5′‐TGATGTGCCAGGGAAGAAAGCCTA‐3'. The mRNA expression level was normalized to beta-Actin and replicated in triplicate according to the manufacturer's instructions. The relative expression quantity was calculated using the 2^-ΔΔCt^ method.

### Western blotting analysis

Cells were collected and lysed using RIPA buffer (50mM Tris, pH 7.4, 150 mM NaCl, 1%TritonX-100, 1% sodium deoxycholate, 0.1% SDS, 2mM sodium pyrophosphate, 25 mM β-glycerophosphate, 1mM EDTA, 1mM Na3VO4 and 0.5 ug/mL leupeptin). Cell lysates were separated by SDS-PAGE, and then electrophoretically transferred to NC membranes (GE Health Care Life Science, Beijing, China). After blocking using 5% nonfat milk in PBST, the membranes were incubated at 4 °C overnight with indicated antibodies. Primary antibodies were from commercial sources: anti-NUS1 (ab155282, Abcam, USA), and anti-Actin (220529 Zen bioscience, Chengdu, China). The secondary antibodies were against rabbit (ab205718, Abcam) and mouse (ab190475, Abcam) were diluted at 1:5000. Membranes were incubated with secondary antibodies for 1 h and were visualized by P-ECL chemiluminescence (CAT: SQ101, EpiZyme, Shanghai, China).

### Cell transfection

Both ccRCC cells A-498 and 786-O cells line were transfected with miR-184-5p mimics (miR10000454-1-5), miR-184-5p inhibitor (miR20000454-1-5), negative control (NC, RiboBio, Guangzhou, China) and NUS1 over expression plasmid using Lipofectamine^TM^ 3000 reagent (Invitrogen) according to the manufacturer's instructions. Both A-498 and 786-O cells were transfected with 50nM miRNAs, 3 µmol/l siRNAs, and 1 µg/µl NUS1 overexpression plasmid (pcDNA3.1-ACSL4 from Shanghai GeneChem Co., Ltd.) using Lipofectamine^TM^ 3000 reagent (Invitrogen) according to the protocols from Invitrogen. Cells were transfected for 24h and then were used to examine the effect of transfection using RT-qPCR. Cells were harvested for further analysis at 24h after transfection.

### Cell viability assay

Human A-498 and 786-O cells line were seeded into 96-well plates at a density of 4×10^3^ cells/well, and cultured in a 5% CO_2_ incubator at 37 °C for 24 h, 48 h, 72 h, and 96 h. Then, 10 μl CCK8 solution was added to each well, and the cells were cultured for 30 min to 4 h according to the manufacturer's instructions. The OD value of the medium was detected using a spectrophotometer at 450-nm wave length.

### Transwell assay

Transwell invasion assays were performed using a 24-well transwell chamber (Greiner bio-one, Switzerland). First, Matrigel (BD Bioscience, USA) was applied to the upper ventricular surface of the Transwell chamber basement membrane, and then 2×10^5^ A-498 and 786-O cells were suspended in 0.2 ml of serum-free medium and were added to the insert. The lower compartment was supplemented with 0.5 ml of medium containing 10% FBS as a chemical attractant. After incubating for 48 hours at 37 °C, the cells on the upper surface of the membrane were carefully removed with a cotton swab and the cells on the lower surface were continuously fixed with 100% methanol and then stained with 0.5% crystal violet. Three random fields of magnification of 200X were selected for each insert, and the number of cells was counted under an optical microscope (Olympus, Japan).

### Cell apoptosis assays

Apoptosis detection assay was performed using Annexin V-FITC Apoptosis Detection Kits (BD, USA) using a FACS analyzer (BD) in accordance with the manufacturer's experiment procedures. After A-498 and 786-O cells were collected and washed in PBS for three times, 500ul cell suspension, 5ul Annexin V-FITC, and 5ul propidium iodide (PI) solution were resuspended in each collection tube. Annexin V-FITC (-) PI (-), E3, stands for normal cells; Annexin V-FITC (+) PI (-), E4, stands for early apoptotic cells; Annexin V-FITC (+) PI (+), E2, stands for late apoptotic cells; Annexin V-FITC (-) PI (+), E1, stands for mechanical necrotic cells. Both early and late apoptotic cell (E4 and E2) were counted and measured as apoptotic cells.

### Cell cycle assay

Flow cytometry was performed to measure the effect of miR-184-5p inhibitor and mimics interference on cell cycle distribution of A-498 and 786-O cells in comparison with NC group. After A-498 and 786-O cells were collected and washed in PBS for three times and disposed using cell cycle assay kit (KeyGen Biotech, Nanjing, China), the percentage of the cells number of each cell cycle phase (G0/G1, S, G2M) was detected using a FACS analyzer (BD, USA).

### Luciferase reporter assay

The online biological databases TargetScan (http://www.targetscan.org/) was used to predict the targets of miRNAs. To investigate whether miR-184-5p could interact with the 3'-UTRs of NUS1, wild-type (WT) 3'-UTR of NUS1 was predicted to interact with miR-184-5p or the mutant (MUT) NUS1 3'-UTR was amplified. A sequence of 3′-UTR of NUS1 containing the predicted miR-184-5p binding site was synthesized and inserted into the p-miR-reporter plasmid (p-miR- NUS1-3′-UTR-WT; Ambion: Thermo Fisher Scientific, Inc., Foster City, CA, USA). Simultaneously, a sequence that contains eight mutant nucleotides of the 3′-UTR of NUS1 was inserted into the p-miR-reporter plasmid (p-miR- NUS1-3′-UTR-MUT). Then, the WT 3'-UTR of NUS1 or MUT 3'-UTR of NUS1, and miR-184-5p mimic were co-transfected into A-498 and 786-O cells by Lipofectamine 3000 according to the manufacturer's instructions. Forty-eight hours after co-transfection, the cells were lysed and assayed using Dual-Luciferase^®^ Reporter Assay Kit (Promega, Madison, WI, USA) based on the manufacturer's instructions.

### Statistical analysis

Survival analysis was performed using transcriptional expression of miR-184-5p and follow-up information were based on 516 patients with ccRCC from TCGA database using Kaplan-Meier methods at Kaplan-Meier Plotter (http://kmplot.com/analysis). Statistical analysis was conducted using GraphPad Prism software (Version 8.0) and the R package (Version 3.6.1). All measurement data are shown as the mean ± standard deviation (SD). Differences in measurement data between groups were analyzed using a two-tailed Student's t-test. Statistical significance was considered at P less than 0.05 for all analyses 17.

## Results

### DEmiRNAs reveals novel role of miR-184-5p in ccRCC

**Figure 1:**
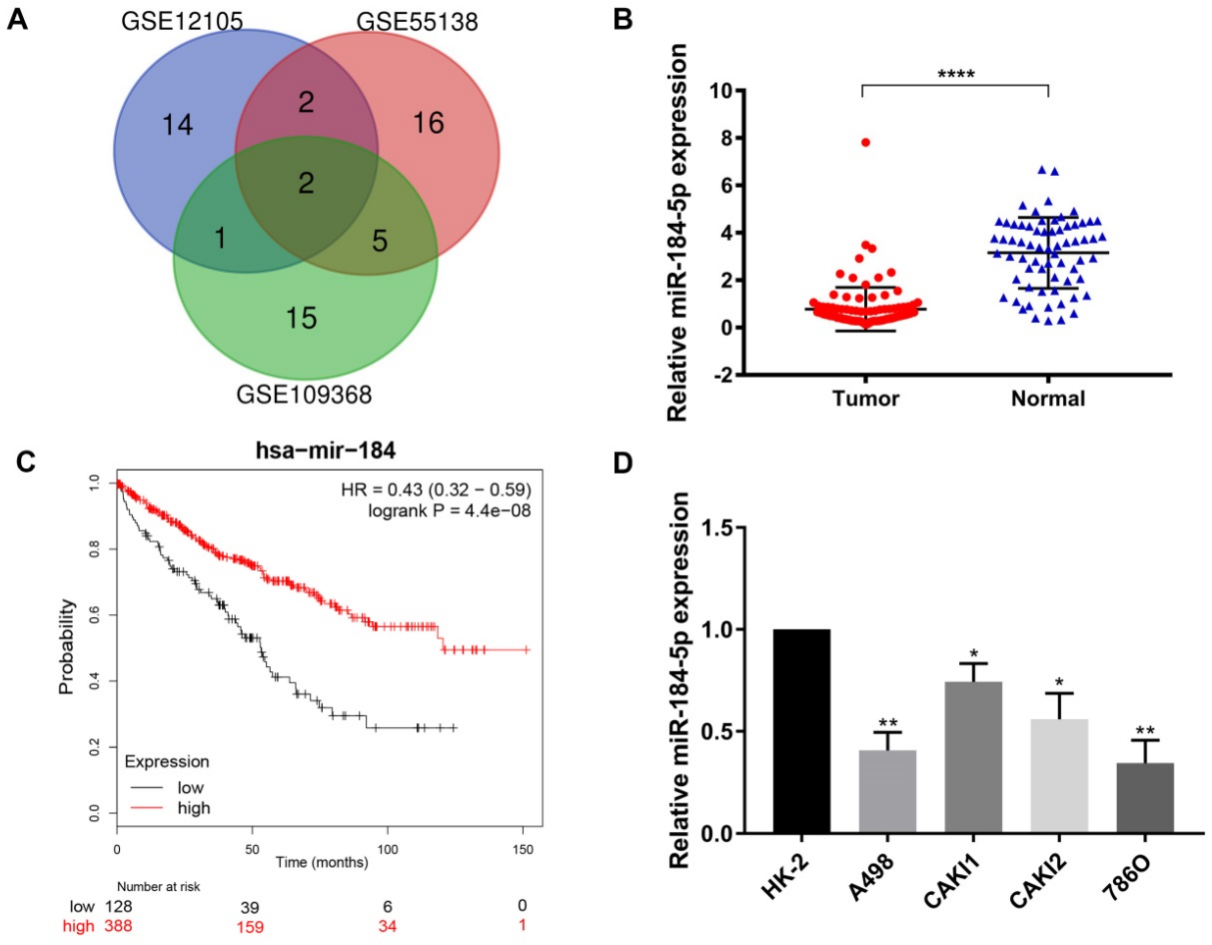
** Screening, identification and prognostic implications of miR-184-5p of ccRCC. (A)** The overlap among the three independent datasets included two significant microRNA, hsa-miR-184-5p and hsa-miR-155, displayed in Venn diagram. (**B**) Relative miR-184-5p expression level is significantly diminished in ccRCC tissues compared with normal tissues base on TCGA-ccRCC cohort (P<0.001). (**C**) Survival curves suggested that decreasing miR-184-5p expression was significantly associated with favorable prognosis for 516 ccRCC patients (P<0.001, HR=0.71). (**D**) miR-184-5p expression level was detected in A-498, CAKI-1, CAKI-2, 786-O ccRCC cell lines and the normal human kidney cell line HK-2 cells. Compared with normal HK-2 cells, the expression level of miR-184-5p was markedly reduced in A-498 cells and 786-O cells, which was thus selected for further subsequent experiments. *p<0.05, **p<0.01, ***p<0.001, ****p<0.0001.

After being normalized and censored of the miRNA microarray information, the DEmiRNAs were determined to be significantly based on the analysis and the statistical parameters after data processing and cleaning. The overlap among the three independent datasets including two significant microRNA, hsa-miR-184-5p and hsa-miR-155, was displayed in Venn diagram (Figure 1A). Then, a total of 533 ccRCC tissues and 72 normal kidney tissues with available transcriptional profiles were enrolled. We found that relative miR-184-5p expression level was significantly decreased in ccRCC tissues compared with normal tissues (P<0.001, Figure 1B) based on TCGA-ccRCC cohort. To explore the potential prognostic value of miR-184-5p in ccRCC, we performed overall survival analysis based on 516 ccRCC patients from TCGA cohort using K-M methods. As is shown in Figure 1C, decreased miR-184-5p expression was significantly associated with favorable prognosis for 516 ccRCC patients (P=4.4e-08, HR=0.43). Subsequently, we detected the miR-184-5p expression level in ccRCC cell lines A-498, CAKI-1, CAKI-2, 786-O and the normal human kidney cell line HK-2 cells. As is illustrated in Figure 1D, compared with normal HK-2 cells, the expression level of miR-184-5p was markedly reduced in A-498 cells and 786-O cells, which was thus selected for further subsequent experiments.

### miR-184-5p regulates proliferation of A-498 and 786-O cells

**Figure 2:**
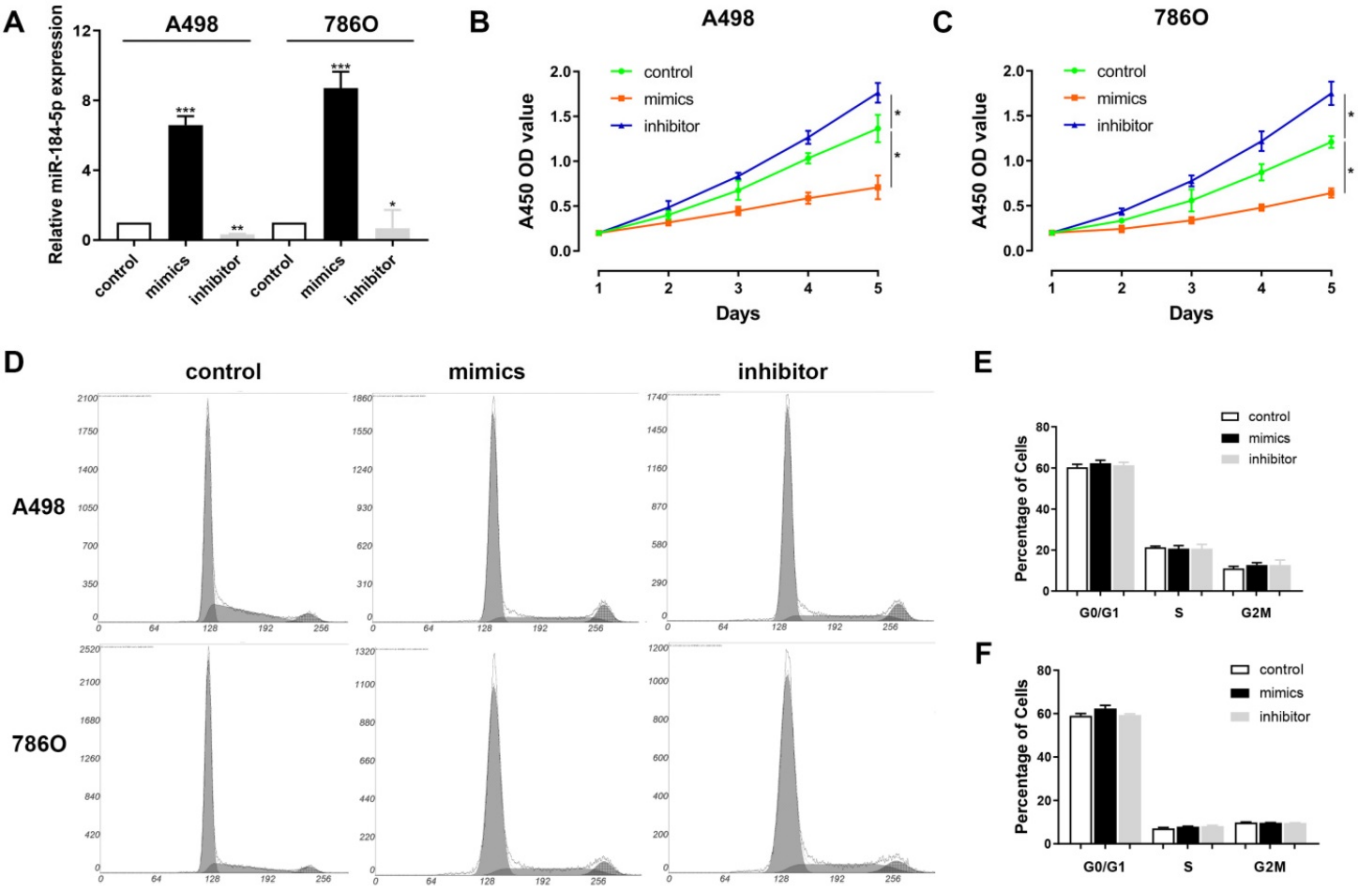
** miR-184-5p regulates proliferation of A-498 and 786-O cells. (A)** Expression level of miR-184-5p was significantly increased in the miR-184-5p mimic-transfecting group cells, and decreased in the miR-184-5p inhibitor-transfecting group in A-498 and 786-O cells (P<0.05).** (B-C)** CCK-8 assay suggested that after A-498 and 786-O cells were up-regulated of miR-184-5p using mimics, the cell growth value of miR-184-5p overexpression group was significantly decreased compared with the negative control group (P<0.01). Conversely, the growth rate of miR-184-5p downregulated cells group markedly increased compared with the negative control group in both A-498 and 786-O cells (P<0.01). (**D-F**) To further measure the effect of miR-184-5p in cell cycle, we transfected mimic and inhibitor of miR-184-5p into A-498 and 786-O cells and found no significant differences in G0/G1, S and G2/M phases compared with the negative control. *p<0.05, **p<0.01, ***p<0.001, ****p<0.0001.

Next, we aimed to explore whether miR-184-5p could modulate the proliferation, of ccRCC cells. Subsequently, we investigated the expression level of miR-184-5p in A-498 and 786-O cells after transfection with mimics, inhibitor or negative control. The results demonstrated that the expression level of miR-184-5p was significantly increased in the miR-184-5p mimic-transfecting group cells, and decreased in the miR-184-5p inhibitor-transfecting group in A-498 and 786-O cells (P<0.05) (Figure 2A). To detect potential function of miR-184-5p, we assessed cell proliferation ability by using a CCK-8 assay after transfecting miR-184-5p mimic or inhibitor into A-498 and 786-O cells. After A-498 and 786-O cells were up-regulated of miR-184-5p using mimics, the cell growth value of miR-184-5p overexpression group was significantly decreased compared with the negative control group (P<0.01). Conversely, the growth rate of miR-184-5p downregulated cells group was markedly increased compared with the negative control group in both A-498 and 786-O cells (P<0.01) (Figure 2B-C). To further measure the effect of miR-184-5p in cell cycle, we transfected mimic and inhibitor of miR-184-5p into A-498 and 786-O cells, and found no significant differences in G0/G1, S and G2/M phases compared with the negative control (Figure 2D-F).

### miR-184-5p regulates invasion and apoptosis abilities of A-498 and 786-O cells

**Figure 3:**
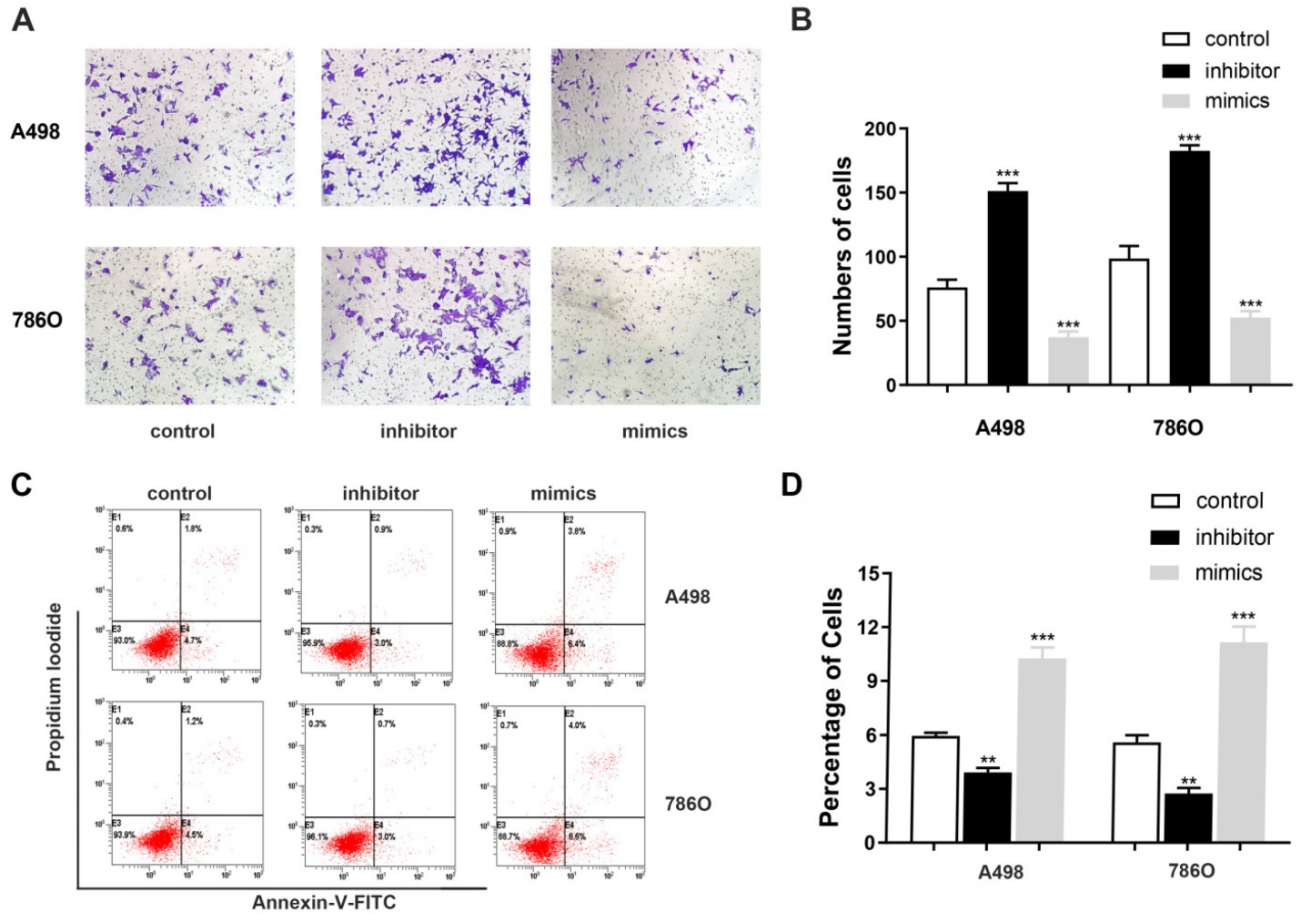
** miR-184-5p regulates invasion and apoptosis of A-498 and 786-O cells. (A-B)** Up-regulation of miR-184-5p could significantly reduce cells invasion capacity. The cell numbers markedly increased after transfection with of miR-184-5p inhibitor (P<0.01) and dropped after transfection with of miR-184-5p mimics (P<0.01), compared with the negative control group. **(C-D)** After transfection with miR-184-5p inhibitor in A-498 and 786-O cells, we found significantly decreased apoptosis cells compared with negative control cell groups measured by propidium iodide (PI) and FITC ‐ Annexin V fluorescence. Annexin V-FITC (-) PI (-), E3, stands for normal cells; Annexin V-FITC (+) PI (-), E4, stands for early apoptotic cells; Annexin V-FITC (+) PI (+), E2, stands for late apoptotic cells; Annexin V-FITC (-) PI (+), E1, stands for mechanical necrotic cells. Both early and late apoptotic cell (E4 and E2) were counted and measured as apoptotic cells. *p<0.05, **p<0.01, ***p<0.001, ****p<0.0001.

Transwell assays were performed to assess malignant ability after transfecting miR-184-5p mimic or inhibitor into A-498 and 786-O cells. The results indicated that up-regulation of miR-184-5p could significantly reduce cells invasion capacity. The cell numbers markedly increased after transfection with of miR-184-5p inhibitor (P<0.01) and dropped after transfection with of miR-184-5p mimics (P<0.01), compared with the negative control group (Figure 3A-B). As is shown in Figure 3C-D, after transfection with miR-184-5p inhibitor in A-498 and 786-O cells, we found significantly decreased apoptosis cells compared with negative control cell groups measured by propidium iodide (PI) and FITC ‐ Annexin V fluorescence. When transfecting into? miR-184-5p mimics, A-498 and 786-O cells markedly increased compared with normal control group (P<0.01).

### miR-184-5p modulates NUS1 expression by directly targeting NUS1-3'-UTR

**Figure 4:**
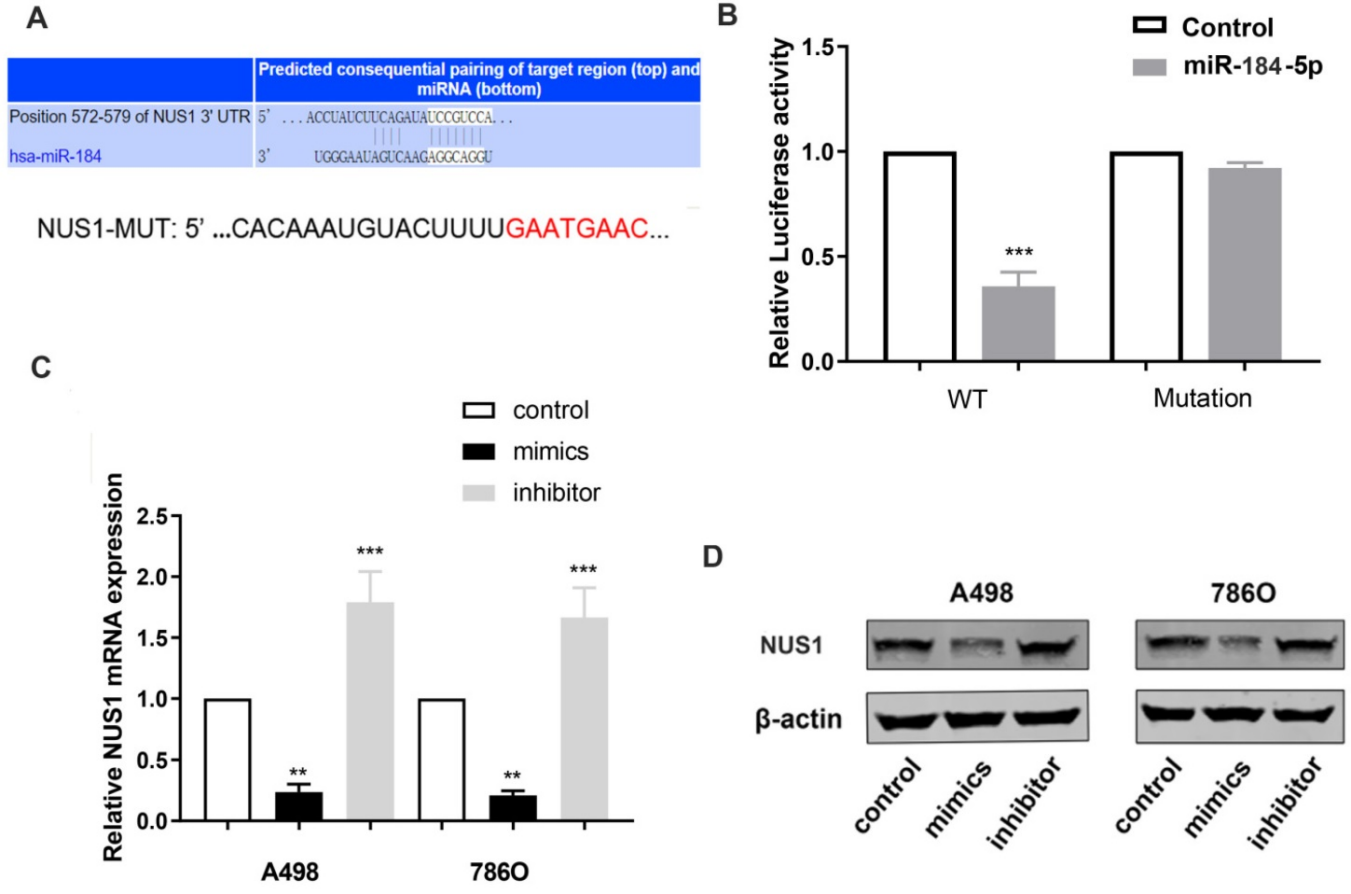
** miR-184-5p modulates NUS1 expression by directly targeting NUS1-3'-UTR. (A)** There is a putative binding site of miR-184-5p in the 3'-UTR of NUS1. **(B)** Activity transfected with miR-184-5p mimic and psiCHECK-WT significantly decreased, while no significant difference was observed in the cotransfection reporter activity with miR-184-5p mimic psiCHECK-Mutant. **(C-D)** Transcriptional and protein expression levels alteration of NUS1 was validated after transfecting miR-184-5p mimic or inhibitor into A-498 and 786-O cells. *p<0.05, **p<0.01, ***p<0.001, ****p<0.0001.

To reveal underlying molecular mechanism of miR-184-5p and potential biological functions in ccRCC cells, TargetScan Human 7.2 (http://www.targetscan.org) was used to search for downstream targets of miR-184-5p. NUS1 was recognized as a potential binding target of miR-184-5p carrying consequential paring of target regions. As is shown in Figure 4A, the 3'-UTR of NUS1 contains a putative binding site for miR-184-5p. Then, we performed a luciferase assay to investigate whether miR-184-5p binds to a putative binding site in the 3'-UTR of NUS1. Overexpression of miR-184-5p decreased the luciferase activity of WT NUS1 3'-UTR (P<0.05, Figure 4B), while miR-184-5p overexpression did not have any influence on the Luciferase activity of MUT NUS1 3'-UTR (Figure 4B). Additionally, we validated mRNA and protein expression level alteration of NUS1 after transfecting miR-184-5p mimic or inhibitor into A-498 and 786-O cells. Significantly decreased expression and increased expression level was observed in mimic and inhibitor groups respectively, suggesting a direct effect of miR-184-5p on NUS1 in A-498 and 786-O cells (Figure 4C-D). Overall, these results showed that miR-184-5p negatively modulates NUS1 protein expression by directly binding to the 3'-UTR of NUS1.

### Enhanced NUS1 expression promotes malignant phenotype of ccRCC cells

**Figure 5:**
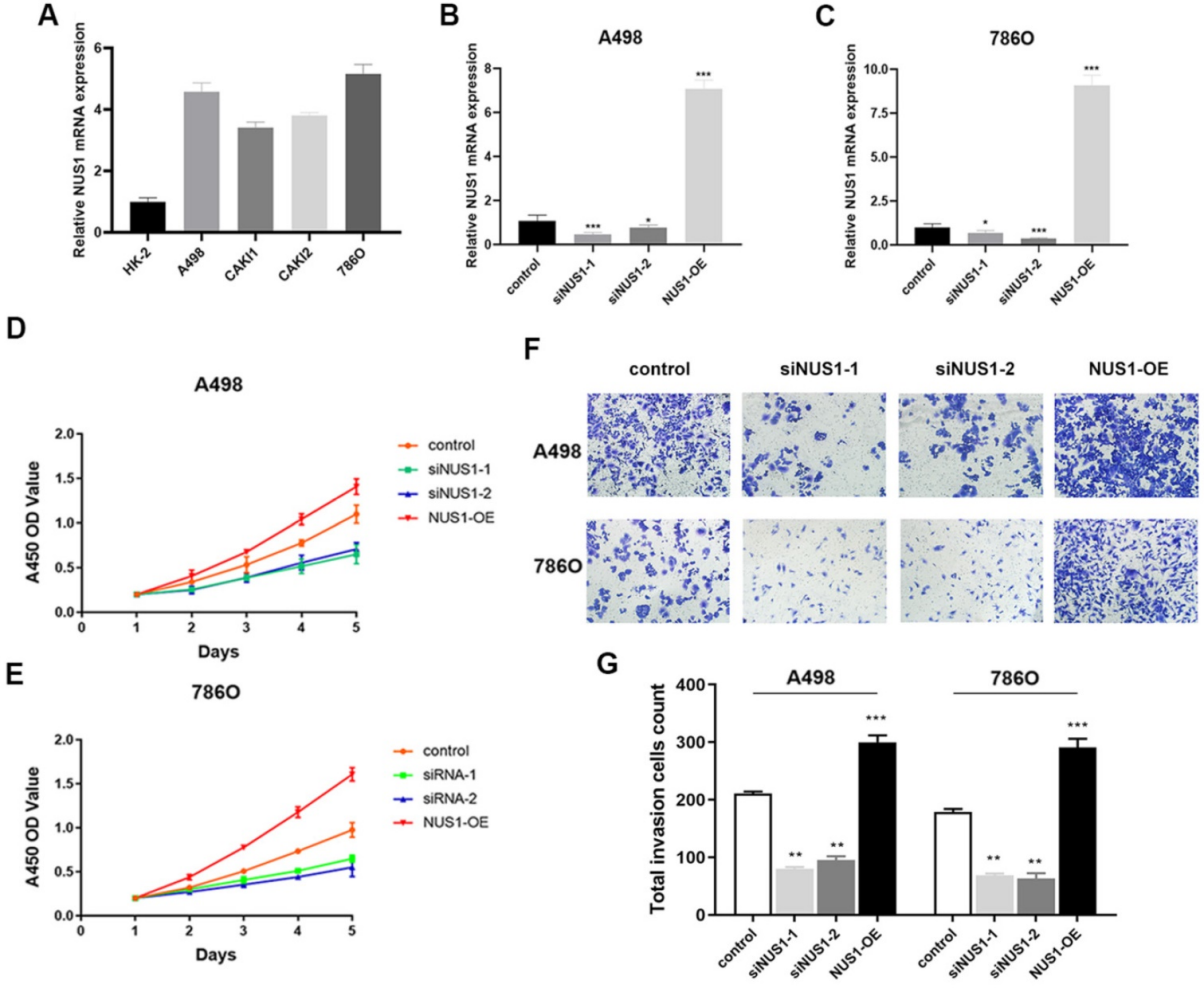
**Enhanced NUS1 expression promotes malignant phenotype of ccRCC cells. (A)** The RT-qPCR showed that NUS1 was up-regulated in four ccRCC cell lines (A-498, CAKI-1, CAKI-2, and 786-O) compared with normal kidney cells (HK-2). **(B-C)** The mRNA and protein expression level of NUS1 in control group, transfecting siNUS1-1 group, transfecting siNUS1-2 group, and transfecting pcDNA3.1-NUS1 group. **(D-E)** The proliferation ability of A-498 and 786-O cells in control group, transfecting siNUS1-1 group, and NUS1 overexpression group. **(F-G)** Representative images of invasion of A-498 and 786-O cells between control group, siNUS1-1 group, and NUS1 overexpression group. **(H)** Quantification analysis of results from (F) and (G). *p<0.05, **p<0.01, ***p<0.001, ****p<0.0001.

NUS1 was shown to be overexpressed in ccRCC and high expression of NUS1 predicted poor prognosis of ccRCC. However, whether NUS1 plays a role in growth and metastasis of ccRCC cells is largely unknown. Therefore, we firstly examined the expression level of NUS1 in several ccRCC cell lines and normal kidney HK-2 cells. Being consistent with the previous studies, the mRNA expression of NUS1 was enhanced in ccRCC cells compared with HK-2 cells (Figure 5A). The relative expression level of NUS1 in these cell lines is opposite to that of miR-184-5p in same cell lines as is shown in Figure 1D. Next, to explore the effect of NUS1 in progression of ccRCC, we transfected two siRNAs specific for NUS1 (siNUS1-1 and siNUS1-2) and pcDNA3.1-NUS1 (NUS1 overexpression plasmid, NUS1-OE) into A-498 and 786-O cells. As is shown in Figure 5B-C, expression of NUS1 was remarkably silenced in A-498 and 786-O cells by siNUS1-1 and siNUS1-2 respectively, and pcDNA3.1- NUS1 markedly enhanced NUS1 expression in both A-498 and 786-O cell lines. The proliferative ability of A-498 and 786-O cells was remarkably elevated in group of NUS1-OE (P<0.01). In contrast, siNUS1 significantly suppressed proliferation of the A-498 and 786-O cells (Figure 5D-E). Consistently, we observed that NUS1 overexpression promoted the migration of A-498 and 786-O cells, while NUS1 silenced by siNUS1 markedly suppressed the migration and invasion of A-498 and 786-O cells (Figure 5F-G). In brief, our results showed that enhanced expression of NUS1 promoted malignant phenotype of ccRCC cells, which is opposite to the effects of miR-184-5p on phenotype of ccRCC.

### NUS1 rescues cells proliferation and migration capacities after miR-184-5p mimic transfection

**Figure 6:**
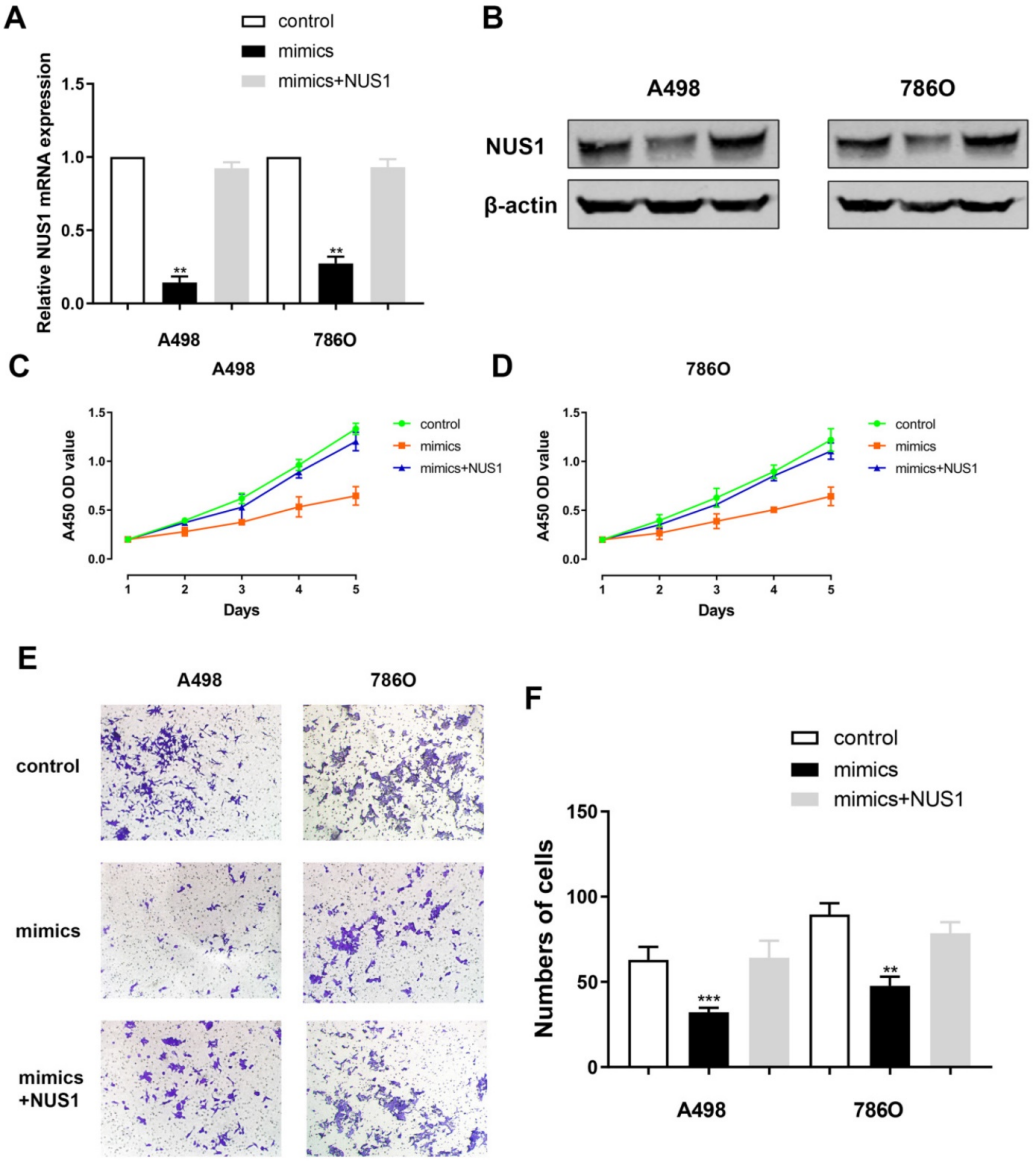
** NUS1 rescues cells proliferation, invasion and migration after miR-184-5p mimic transfection. (A-B)** Significant decrease of NUS1 mRNA and protein expression level were found after transfecting mimics, while relative expression was rescued to normal when exposed to NUS1 compared with negative control group. **(C-D)** Cell proliferation was significantly decreased in miR-184-5p upregulation group, while cell numbers were rescued to the similar as the normal control group. **(E-F)** Invasion and migration assay showed that cell invasion vitality was significantly decreased in miR-184-5p upregulation mimic group, while cell numbers were rescued to normal as the negative control group in A-498 and 786-O cells. *p<0.05, **p<0.01, ***p<0.001, ****p<0.0001.

Relative NUS1 mRNA and protein expression level alteration was detected after transfecting miR-184-5p mimic or transfecting miR-184-5p mimic combined with NUS1 compared with negative control. It suggested a significant reduction of NUS1 mRNA and protein expression level after transfecting mimics while relative expression was rescued to normal when exposed to NUS1 compared with negative control group (Figure 6A-B). Subsequently, to explore whether miR-184-5p inhibits proliferation of A-498 and 786-O cells by directly targeting NUS1, we assessed CCK-8 assay after transfecting miR-184-5p mimics or miR-184-5p mimics combined with NUS1 into A-498 and 786-O cells, compared with negative control. It suggested that cell proliferation was significantly decreased in miR-184-5p upregulation group, while cell numbers were rescued to the similar one as the negative control group (Figure 6C-D). In addition, results of invasion and migration assay showed that cell migration vitality was significantly decreased in miR-184-5p mimic group, while cell numbers were rescued to normal as the negative control group in A-498 and 786-O cells showed (Figure 6E-F).

## Discussion

Extensive research finds that many miRNAs play key roles in various cancer types such as kidney cancer 18, liver cancer 19, colorectal cancer 20, breast cancer 21, and head and neck cancer 22. Interestingly, based on sequencing data, Zhao et al. provided a two-dimensional map of the mRNA and miRNA expression profiles of ccRCC and found that miR-210, miR-184 and miR-206, play important roles in ccRCC development and serve as a promising novel candidate of ccRCC 23, 24. Previous studies also found that up-regulation of pre-miR-184 altered the metabolic reprogramming and proliferation characterizations of ccRCC by regulating cell glycolysis effects 25. Besides, markedly up-regulated miR-184 was found in RCC cells, which could inhibit cell apoptosis and facilitate tumor proliferation and migration capacities through modulating β-catenin/TCF4 pathway or silencing LINC01094 inhibited SLC2A3 expression 26, 27.

In this study, we first detected miR-184-5p expression in human ccRCC tissues and cells, and then confirmed that miR-184 expression in human ccRCC cells and tissues was reduced. In addition, we performed CCK-8 analysis, invasion and migration analysis, cell apoptosis and cell cycle analysis to explore the biological role of miR-184-5p. Meanwhile, up-regulating miR-184-5p by transfection with miR-184-5p mimics can significantly inhibit A-498 and 786-O cell proliferation, migration, and invasion capabilities. Further Luciferase reporter detection revealed that NUS1 is a direct target of miR-184-5p. Finally, NUS1 rescues cells proliferation, migration and apoptosis capacity after miR-184-5p mimic transfection. Similarly, miR-184 plays a role in inhibiting the growth and invasion of cancer cells in tumors. For example, the suppression of miR-184 could upregulate SND1 and contribute to tumor invasion in malignant glioma 28. Another study indicated that the knockdown of miR-184 dramatically promoted neuroblastoma cell proliferation, increasing the levels of AKT2 and EMT process 29. Conversely, previous studies also demonstrated pro-tumorigenesis feature of miR-184 in cancers. In 2018, Chen et al. found that miR-184 could regulate the proliferation of the tongue squamous cell carcinoma by targeting SOX7 30, 31. Overall, these studies indicate a possible role of miR-184 in modulating tumor progression.

As the most common renal malignancies, ccRCC accounting for approximately 3% of adult cancer 32, 33. The existence of intratumor genetic heterogeneity in ccRCC has not been fully delineated, and the heterogeneity was also reflected in the expression of tumor markers. In this study, we firstly detected that miR-184-5p regulates the proliferation and migration of renal cancer cells by directly targeting NUS1. NUS1 Dehydrodolichyl Diphosphate Synthase Subunit (NUS1), also known as Nogo-B Receptor, along with DHDDS, forms the dehydrodolichyl diphosphate synthase (DDS) complex, an essential component of the dolichol monophosphate (Dol-P) biosynthetic machinery. Both subunits contribute to enzymatic activity, i.e., condensation of multiple copies of isopentenyl pyrophosphate (IPP) to farnesyl pyrophosphate (FPP) to produce dehydrodolichyl diphosphate (Dedol-PP), a precursor of dolichol phosphate which is utilized as a sugar carrier in protein glycosylation in the endoplasmic reticulum (ER) 34-36. It also regulates the glycosylation and stability of nascent NPC2, thereby promoting trafficking of LDL-derived cholesterol. Meanwhile, NUS1 has been widely documented to regulate cell growth, adhesion, and differentiation 37. In addition, there is growing evidence that NUS1 is upregulated in several cancer types, such as breast cancer 38, liver cancer 39, 40, and lung cancer 41. But the relationship between NUS1 and kidney cancer has not been fully elucidated. Our research also confirms that NUS1 can rescue cell proliferation, invasion and migration inhibition caused by miRNA-184-5p overexpression. Understanding of the mechanism of NUS1 on the development of ccRCC provided a new insight into the modulation of NUS1 on the development of ccRCC and the results supported NUS1-based therapy for ccRCC patients.

miR-184-5p has anti-tumor effects in certain cancers, such as glioma 42, colorectal cancer 43 and non-small cell lung cancer 44. In addition, miR-184 inhibits tumor migration and metastasis in nasopharyngeal carcinoma by Targeting Notch2 45. However, the miR‐184-5p prognostic relevance and effects in ccRCC are not clear. In the current studies, these results indicated that miR‐184-5p could serve as an independent prognostic biomarker and potential treatment target for ccRCC via directly targeting NUS1. However, since miR-184-5p has multiple potential target genes, we only explored the 3'-UTR targeting NUS1 based on literature and experimental verification and neglect possible pleiotropic effects, which may reduce the conviction of this study.

## Conclusion

In summary, this result indicates that decreased miR-184-5p expression in human ccRCC cell lines and tissues, while up-regulation of miR-184-5p can at least partially regulate the proliferation, invasion and apoptosis capacity of ccRCC cells by directly targeting NUS1. These findings provide new insights into the regulation of ccRCC development by miR-184-5p, and the results support miR-184-5p-based therapy for ccRCC patients.

## Abbreviations

ccRCC: clear cell renal cell carcinoma
DEmiRNAs: differential expressed miRNAs
GEO: Gene Expression Omnibus database
OS: overall survival
TCGA: The Cancer Genome Atlas
NUS1: NUS1 dehydrodolichyl diphosphate synthase subunit
